# Supplementary material for: Learning curve and surgical outcome of robotic assisted colorectal surgery with ERAS program
Source: Sci Rep. 2022 Nov 29;12:20566. doi: 10.1038/s41598-022-24665-w (PMC9709162; doi:10.1038/s41598-022-24665-w)
Supplement: Supplementary file 1 — Supplementary Information 1. [file 41598_2022_24665_MOESM1_ESM.zip › LSC Raw data-Ñ[▒K/135/P_ñw╝╨░O▒Kñσ.pdf]

[redacted]

Pathologic diagnosis:
1. Rectum, laparoscopic low anterior resection --- a. Adenocarcinoma, moderately differentiated. Tumor invaded to mesocolic soft tissue. The bilateral cut ends and mesocolic lymph nodes (0/16) are free of tumor b. Tubular adenoma.
2. IMA root, dissection --- Unremarkable fibroadipose tissue and lymph nodes (0/3).
3. Rectum, cut end, distal, resection --- Unremarkable.
4. Liver, S5, biopsy --- Metastatic adenocarcinoma, favored colonrectal origin.
5. Liver, S6, biopsy --- Metastatic adenocarcinoma, favored colonrectal origin. The section margin is 1 mm to tumor.

Ancillary study for diagnosis:
1. IHC stains for SATB2 (+) done on section C.
2. IHC stains for PMS2 and MSH6 are done on section C. Both markers are preserved.

Prognostic and predictive factor:
1. Tumor size: 4x3.5 cm.
2. Depth of invasion: Pericolorectal tissue.
3. Histologic grade: G2: Moderately differentiated.
4. Angiolymphatic invasion: Not identified.
5. Perineural invasion: Present.
6. Tumor deposits (discontinuous extramural extension): Present, specify number of deposits: 1.
7. Macroscopic tumor perforation: Not identified.
8. Surgical Margins: a) Proximal Margin: Uninvolved by invasive carcinoma, distance of tumor from margin: 15 cm. b) Distal Margin: Uninvolved by invasive carcinoma, distance of tumor from margin: 1.8 cm. c) Radial or Mesenteric Margin: Involved by invasive carcinoma (tumor present 0-1 mm from margin).
9. Lymph node status: Uninvolved (0/19).
10. Extranodal involvement: Not identified.
11. Tumor regression grading S/P CCRT: No known presurgical therapy.
12. Type of polyp in which invasive carcinoma arose: Not identified.
13. Additional pathologic findings: Adenoma.
14. Pathological TNM Stage: pT3N1cM1a(According to the eighth edition, American Joint Committee on Cancer Staging Guidelines for Tumors).
15. TNM descriptors: Not applicable.

Gross description:
The specimen consists of 1) a segment of rectum, 21.0 cm in length and 7.0 cm in proximal cut end circumference, 5.0 cm in distal circumference, and perirectal soft tissue. A gray tan firm annular elevated and centrally ulcerative tumor, 4.0x3.5 cm, is noted, 1.8 cm to the proximal cut end. On cross section, the tumor invades to the perirectal soft tissue with 3 mm to the radial margin, and serosal retraction is also noted. A polyp, 0.4x0.3x0.3 cm, 8 cm to the proximal cut end, is seen. The both cut ends are free of tumor grossly. 2) IMA root, 3.8x3.2x1.0 a piece of tan yellow soft tissue, cm. 3) distal cut end, a piece of tan mucosa-covered soft tissue, 2.2x1.5x0.5 cm. 4) liver S5, 7.0x7.0x2.4 cm, the specimen has been cut when received, a yellow tan nodule, 0.8x0.8x0.5 cm, is noted. 4) liver S6, 6.2x5.4x1.3 cm. A yellow tan nodule, 2.8x2.0x1.4 cm, is noted when serial cut. Representative part for section: A) distal cut end B) proximal cut end C-D) tumor E) polyp F) non tumor rectum G) specimen 2 H) specimen 3 J) specimen 4 K) specimen 5 L-N) lymph nodes.

Note:
1. The interpretation of MMR panel and recommended studies are as below: (1) No loss of nuclear expression of MMR proteins: low probability of microsatellite instability-high (MSI-H) . (2) Loss of nuclear expression of MLH1 and PMS2: testing for methylation of the MLH1 promoter and/or mutation of BRAF is indicated (the presence of a BRAF V600E mutation and/or MLH1 methylation suggests that the tumor [illegible]

is sporadic and germline evaluation is probably not indicated; absence of both MLH1 methylation and of BRAF V600E mutation suggests the possibility of Lynch syndrome, and sequencing and/or large deletion/duplication testing of germline MLH1 may be indicated). (3) Loss of nuclear expression of MSH2 and MSH6: high probability of Lynch syndrome (sequencing and/or large deletion/duplication testing of germline MSH2 may be indicated, and, if negative, sequencing and/or large deletion/duplication testing of germline MSH6 may be indicated). (4) Loss of nuclear expression of MSH6 only: high probability of Lynch syndrome (sequencing and/or large deletion/duplication testing of germline MSH6 may be indicated). (5) Loss of nuclear expression of PMS2 only: high probability of Lynch syndrome (sequencing and/or large deletion/duplication testing of germline PMS2 may be indicated). (6) Please note: There are exceptions to the above IHC interpretations. These results should not be considered in isolation, and clinical correlation with genetic counseling is recommended to assess the need for germline testing.

#T-59300_2 #M-81403_2 2 1105 S0000F
#T-62000_2 #M-81406_2 2 1104 000000
#T-C4560_2 #M-09183_2 0 1105 000000

已電子簽章

敬告：本單僅供醫師參考使用，不具任何申請效力！！
請病患妥為保管，若不慎遺失需自負個人資料外洩責任！
